# Petroleum in Phthisis

**Published:** 1881-02

**Authors:** 


Petroleum in Phthisis.—This article has been favorably spoken of as being of great and unexpected value in consumption, in teaspoon-doses three or four times daily. Almost every article of medicine has strutted its hour upon the stage as a consumption cure, before the profession, within a dozen years; and it may be that coal oil is destined to follow the fate of its predecessors, sooner or later; but some how, we incline to the belief that it is destined to take a higher stand than any of the vaunted remedies in this great destroyer of the races. Will not some of our readers give it a trial and report the results to us?

